# Formation of Nanocomposites by Oxidizing Annealing of SiO_*x*_ and SiO_*x*_<Er,F> Films: Ellipsometry and FTIR Analysis

**DOI:** 10.1186/s11671-015-0933-0

**Published:** 2015-05-27

**Authors:** Mykola V Sopinskyy, Natalya A Vlasenko, Igor P Lisovskyy, Sergii O Zlobin, Zinoviia F Tsybrii, Lyudmyla I Veligura

**Affiliations:** V. Lashkaryov Institute of Semiconductor Physics, National Academy of Sciences of Ukraine, 45, Prospect Nauky, 03028 Kyiv, Ukraine

**Keywords:** Nanocomposite, Silicon nanoparticles, Silicon oxide films, SiO_*x*_, Erbium, Fluorine, ErF_3_, Infrared spectroscopy, Ellipsometry, 33.20.Ea, 33.50.Dq

## Abstract

The structural-phase transformations induced by air annealing of SiO_*x*_ and SiO_*x*_ < Er,F > films were studied by the combined use of infrared spectroscopy and ellipsometry. The films were prepared using vacuum evaporation of SiO powder and co-evaporation of SiO and ErF_3_ powders. The annealing took place at moderate temperatures (750 and 1000 °C). It was found that the micro- and macrostructure of the annealed films is similar to the structure of the Si–SiO_*x*_ nanocomposites obtained by annealing SiO_*x*_ in vacuum or inert atmosphere and subjected to post-annealing in oxidizing atmosphere. This proves that the phase separation in the non-stoichiometric SiO_*x*_ films proceeds much faster than their oxidation. The results of the work point at a possibility to simplify the annealing technology by replacing the two-step annealing with one-step in the oxygen-containing environment while maintaining the positive effects. The differences in the structure of the nanocomposites obtained by annealing the SiO_*x*_ and SiO_*x*_ < Er,F > films are explained by the action of Er centers as the promoters for SiO_*x*_ disproportionation, as well as the enhanced action of F on the processes of disorder-to-order transition and crystallization in amorphous silicon.

## Background

Thin-film nanocomposites consisting of crystalline and amorphous Si nanoparticles embedded in silicon oxide layers have been actively studied during the last two decades as suitable materials for non-volatile memory devices, third generation of photovoltaic, and other applications. An undiminishing interest in such composites (including those doped with Er) continues because of the possibility to fabricate Si-based light-emitting sources for optoelectronic devices [1, 2].

One of the most widespread methods to produce these composites is the thermally stimulated phase separation in non-stoichiometric silicon oxide films (SiO_*x*_, *x* < 2). Such a process has been studied in detail [1]; however, the investigations were mainly concerned with the study of structural-phase transformations caused by thermal annealing of SiO_*x*_ films in vacuum or in inert atmosphere. At the same time, a number of studies examined the possibility to improve the performance of nanocomposite films by additional heat treatments in the oxygen environment [3–7]. It was shown, in particular, that such a post-annealing (including that in air atmosphere [6]) allows to increase the luminescence efficiency due to good passivation of silicon dangling bonds on the surface of nanoparticles with oxygen atoms [3–6] and the elimination of some active impurities (like hydrogen), which may be the reason of device instability under some circumstances (elevated temperatures, irradiation, etc.). Moreover, oxidation makes it possible to reduce the size of nanoparticles, changing to some extent the spectrum of their light emission. The processes of silicon nanoparticle formation and oxidation may be superimposed if silicon oxide films are subjected to high-temperature treatment in oxygen-containing environment. In that way, it is possible to move from the two-step to one-step technology. However, such a method requires careful study, taking into account the fact that the phase separation in SiO_*x*_ films and their oxidation are competing processes [8].

Recently, we have produced the luminescent systems using high-temperature thermal treatments of SiO_*x*_ and SiO_*x*_ < Er,F > films in the air [9]. The presence of silicon nanoparticles (both amorphous and crystalline) in them has been confirmed by Raman spectroscopy [9, 10]. The detailed data both on silicon inclusions and oxide matrix properties are necessary for subsequent performance optimization of these nanostructures. Earlier, under investigation of SiO_*x*_ films annealed in vacuum [11], it was shown that the combination of infrared spectroscopy and ellipsometry can provide available information on the composition, homogeneity, and structural arrangement of the matrix as well as Si volume fraction and the morphology of Si nanoparticles. Hence, the purpose of this paper is to spread such an approach onto investigation of SiO_*x*_ and SiO_*x*_ < Er,F > films exposed to heat treatment in the air at the temperatures of 750 and 1000 °C.

## Methods

SiO_*x*_ and SiO_*x*_ < Er,F > films for the ellipsometric and Fourier transform infrared spectroscopy (FTIR) characterization were deposited on both side polished c-Si (2 × 2 cm^2^) substrates using evaporation of SiO powder (Cerac Inc., purity of 99.9 %) and co-evaporation of SiO and ErF_3_ powders, respectively, in 10^−3^ Pa vacuum. At the same time, the films were deposited on both side polished (2 × 2 cm^2^) silica substrates. It allows to estimate the value of the absorption index of the films at wavelength *λ* = 632.8 nm from the measurements of the transmission spectra in the visible and near-IR range of spectrum. (The detailed consideration of the transmission spectra for the deposited and annealed films will be reported separately).

Due to oxidation effect in residual gas (molecules of water vapor, oxygen, carbon monoxide, carbon dioxide, etc.), the value of stoichiometry index (*x*) in the as-deposited films was larger than that in the evaporated material and was about 1.4. ErF_3_ concentration was ~1 mol %. To produce uniform thickness of as-deposited film over the surface of all the substrates, they were placed on a carousel rotating at 30 rpm. The average deposition rate was ~0.6 nm/s. The thickness value of the as-deposited films was ~1 μm. To provide a better adhesion during deposition, the substrate was heated—its temperature was maintained at 150 °C. After deposition, the samples were subjected to 1-h annealing in air at temperature *Т*_an_ = 750 and 1000 °C.

Infrared spectra of the SiO_*x*_/Si and SiO_*x*_ < Er,F>/Si samples were measured using single-beam FTIR PerkinElmer Spectrum BXII spectrometer working in the transmission mode at the normal incidence. The accuracy of the wave number value determination was ~0.5 cm^−1^. The accuracy of IR intensity measurements within the region of Si–O absorption band was about ±1 %. To avoid the influence of silicon substrate and native oxide layer during measurements, the part of substrate was used as reference sample, i.e., the absorbance spectra of the oxide films under investigation were obtained by subtracting the absorbance spectrum of the reference sample from the total signal. Additionally, the baselines of the resulting spectra have been corrected. To minimize an influence of atmosphere, all FTIR measurements have been carried out in dessicated atmosphere with low content of water vapor; the measurement of the reference Si substrate sample spectrum was carried out immediately after the measurement of the spectrum of each film/Si sample. The main absorption band for SiO_*x*_ corresponding to the asymmetric stretching vibrations of the bridging oxygen atoms (maximum position lies within the range of ~1000–1100 cm^−1^ depending on the oxygen content) has been investigated. This band was deconvoluted into Gaussian profiles, whose maximum positions are connected with a certain type of oxygen atom structural arrangement in the oxide network, i.e., with Si–O_*y*_–Si_4 − *y*_ (1 ≤ *y* ≤ 4) molecular complexes (in the case of SiO_*x*_ matrix) or with different kinds of SiO_4_ tetrahedra rings (in the case of SiO_2_ matrix). The existence of such a bulk structural component is postulated by random bonding model (RBM). Their portion has been experimentally estimated using the intensity of corresponding IR spectra contributions according to the approach that was previously suggested [12, 13]. The purpose of the analysis was to establish the changes in the content of structural components as the result of annealing. The accuracy of the deconvolution procedure was characterized by the standard deviation of the Gaussian sum from the experimental curve. In our experiments, this deviation did not exceed 10^−2^.

The films were also analyzed by monochromatic multi-angle ellipsometry (*λ* = 632.8 nm) using LEF-3 M-1 ellipsometer and in-house developed software modeling optical characteristics of thin-film structures (including sharp or smooth optical non-uniformity over depth and anisotropy) [14, 15]. The determination of quantitative values for the parameters that characterize these properties was achieved by solving the inverse task of ellipsometry (ITE): the true values of models’ parameters were assumed to be the ones minimizing the mean squared error (MSE):

$$ \mathrm{M}\mathrm{S}\mathrm{E} = \sum \left[{\left\{{\varPsi}^{\exp}\left({\varphi}_{0i}\right)\hbox{--} {\varPsi}^{\mod{}}\left({\varphi}_{0i}\right)\right\}}^2+{\left\{{\Delta}^{\exp}\left({\varphi}_{0i}\right)\hbox{--} {\Delta}^{\mod{}}\left({\varphi}_{0i}\right)\right\}}^2\right], $$

where *Ψ*^exp^(*φ*_*0i*_) and Δ^exp^(*φ*_*0i*_) are experimentally measured values of polarization angles *Ψ* and Δ, respectively, for 11 incidence angles *φ*_*0i*_ from the range of 45°–70° and *Ψ*^mod^(*φ*_*0i*_) and Δ^mod^(*φ*_*0i*_) are calculated for the same incidence angles using the adopted model. Besides the minimum of MSE, the important model adequacy evaluation is the physical basis of the obtained solution, “shrinking” of the more complex models to a simpler model, and the match of the absorption index values obtained ellipsometrically and photometrically.

As it will be seen below, when presenting the results of the simulation, we used single-layer and two-layer models with the following types of layers:

- Isotropic uniform transparent layer (IUTL) with *n*, *h*.
- Isotropic uniform absorbing layer (IUAL) with *n*, *k*, *h*.
- Uniaxially anisotropic uniform absorbing layer (UAUAL) with *n*_o_, *n*_e_, *k*_o_, *k*_e_, *h*.
- Isotropic linearly non-uniform transparent layer (ILNUTL) with *n*_b_, *n*_t_, *h*.
- Isotropic linearly non-uniform absorbing layer (ILNUAL) with *n*_b_, *n*_t_, *k*_b_, *k*_t_, *h*.
- Uniaxially anisotropic linearly non-uniform transparent layer (UALNUTL) with *n*_ob_, *n*_eb_, *n*_ot_, *n*_et_, *h*. Here, *h* is the layer thickness and *n* and *k* are the refractive and absorption indexes, respectively. Lower subscript letters denote the following: o, ordinary; e, extraordinary; b, bottom; and t, top. The numbering of the layers is from the substrate.

## Results

**Fig. 1:**
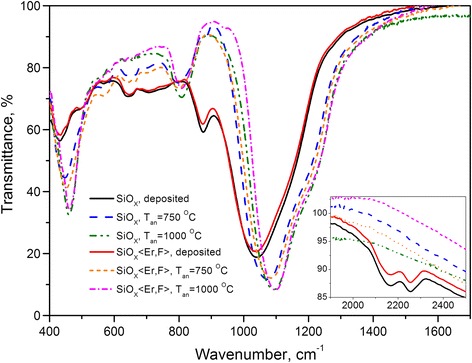
IR transmission spectra of the as-deposited and air-annealed SiO_*x*_ and SiO_*x*_ < Er,F > films

**Fig. 2:**
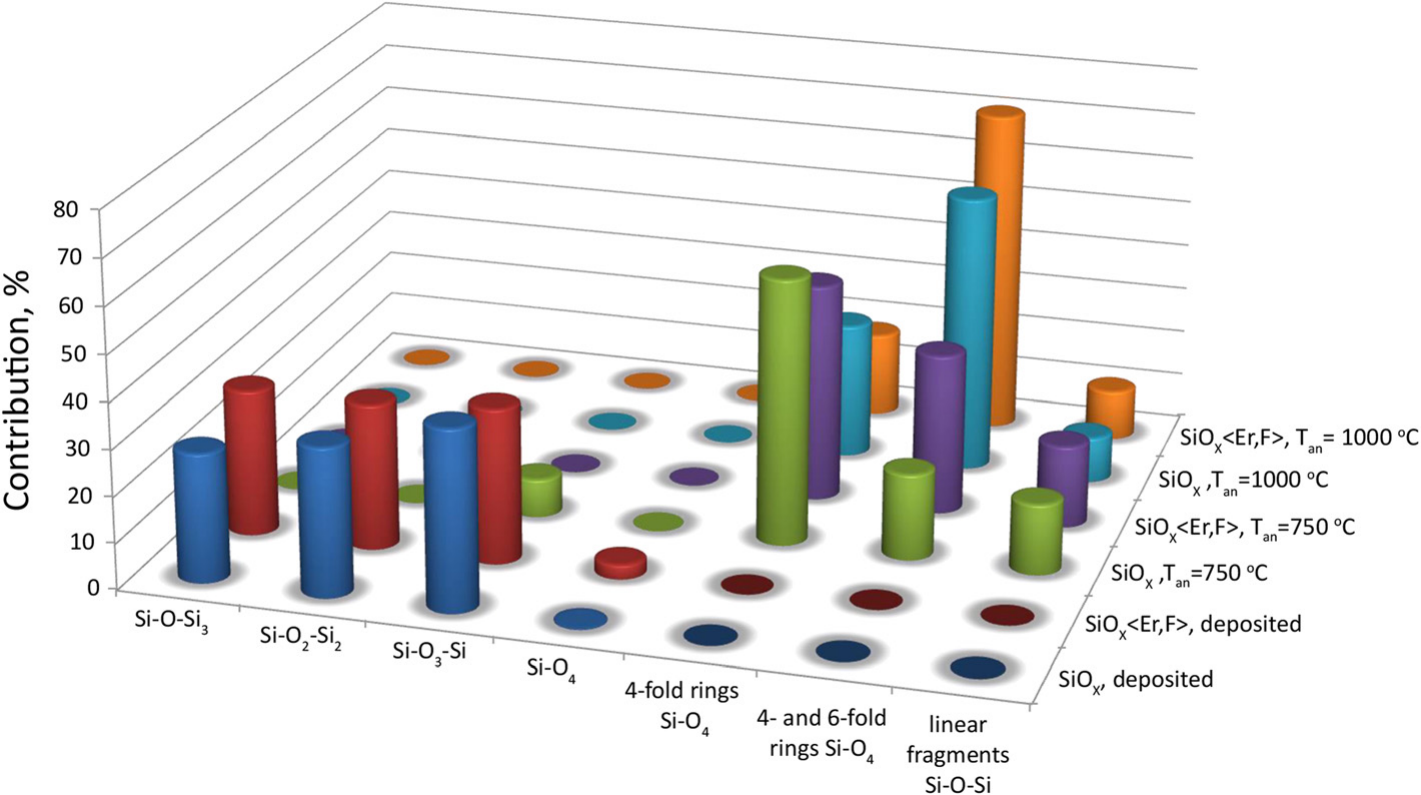
The contribution of the sub-bands corresponding to different structural complexes into the main Si–O absorption band for the as-deposited and air-annealed SiO_*x*_ and SiO_*x*_ < Er,F > films

The main transmission band of the deposited SiO_*x*_ and SiO_*x*_ < Er,F > films had the typical shape with the minimum at *ν*_M_ ≈ 1040 cm^−1^ (Fig. 1). This value allowed us to estimate the value of stoichiometry index to be *х* ≈ 1.45 [16]. Deconvolution of the absorbance band and its analysis showed that the structure of initial oxides is described as a mixture of silicon-oxygen molecular complexes Si–O*y*–Si_4 − *y*_ (1 ≤ *y* ≤ 4) (Fig. 2). Also, the SiH hydrides are present in the oxide network, mainly in the form of О_3_–Si–Н and O_2_–Si–H_2_ or O–Si_2_–H_2_ complexes [17]. It is confirmed by the presence of absorption bands at ~876, ~2155, and ~2255 сm^−1^ (Fig. 1).

When solving the ITE for every sample, we started with the simplest model, the IUTL model, which is characterized by two parameters, the refractive index *n* and the thickness *h*. For the as-deposited SiO_*x*_ film, the IUTL model gives:

- MSE_min_ *=* 12.78 deg^2^ at *n* = 1.712, *h =* 1040.4 nm.

The three-parametric models give:

- MSE_min_ = 7.69 deg^2^ at *n*_b_ =1.6144, *n*_t_ =1.769, *h* = 1055 nm;
- MSE_min_ *=* 2.45 deg^2^ at *n* = 1.708, *k* = −0.0021, *h =* 1043 nm;
- MSE_min_ *=* 0.71 deg^2^ at *n*_o_ = 1.7178, *n*_e_/*n*_o_ = 1.011, *h* = 1030 nm.

From the consideration of the four simplest models, we can already conclude that the most appropriate model for the as-deposited SiO_*x*_ film is the model of anisotropic layer. This is also confirmed by the expansion of both non-uniform and anisotropic models by including the absorption. In the frameworks of these five-parametric models, the following are obtained:

- MSE_min_ = 0.30 deg^2^ at *n*_b_ =1.821, *n*_t_ =1.635, *k*_b_ = 0.0065, *k*_t_ = −0.0143 (*k*_av_ = −0.004), *h* = 1027 nm.
- MSE_min_ *=* 0.0794 deg^2^ at *n*o = 1.706, *n*_e_/*n*_o_ = 1.0065, *k*_о_ = −0.002, *k*_е_ = 0.003 (*k*_av_ = −0.0003), *h* = 1027 nm.

The value *k*_о_ = 0.0018 has been obtained for the as-deposited SiO_*x*_ film from the measurements of the transmission spectra in the visible and near-IR range of the spectrum. It is seen that the value of the absorption coefficient for the latter model is closer to the photometrically measured one. The fact that *k*_*o*_ has negative (although very small) value indicates an incomplete adequacy of the UAUAL model. This means that not all the factors affecting the optical response of the “silicon substrate–as-deposited SiO_*x*_ film” sample are taken into account. Apparently, the in-depth non-uniformity of the film also makes a certain contribution to its optical response. Nevertheless, the difference from the *k*о photometric value is only ≈ 0.004, which should be considered as rather decent match.

The same algorithm of solving ITE has shown that the as-deposited SiO_*x*_ < Er,F > film is well described by the UAUAL model at *n*_o_ = 1.664, *n*_e_/*n*_o_ = 1.0084, *k*_o_ = 0.002, *k*_e_ = 0.006, *h* = 1035 nm (the value *k*_о_ = 0.0024 has been obtained for this film from photometric measurements). The UAUAL model shows both the positive birefringence (*n*_e_ > *n*_o_) and positive dichroism (*k*_e_ > *k*_o_) both in the as-deposited SiO_*x*_ and SiO_*x*_ < Er,F > films. Positive optical anisotropy (*n*_e_ > *n*_o_) in films is often associated with the columnar porous structure with the preferred orientation of columns and pores along the normal to the substrate [18].

Thermal treatments of the films in the air at the *T*_an_ of 750 and 1000 °C led to substantial change in their composition and structure. First, in the FTIR spectra, the peaks connected with hydrogen disappeared (Fig. 1), most likely because of hydrogen desorption from the films. Second, the new band at ~810 cm^−1^ appeared (Fig. 1). Third, Si–O absorption band has shifted to high-frequency region, and its intensity and shape have remarkably changed (Fig. 1). The degree of the changes depends on both the temperature and the presence of Er and F impurities. The presence of impurities enhances the decrease of Si–O absorption band width at half maximum and the shift of the absorption maximum position as the result of annealing. This influence is not random but monotonic, being especially pronounced for samples annealed at 1000 °C. Finally, the treated films cannot be satisfactorily described by any single-layer ellipsometric model.

After annealing at *750 °С*, the shift of the main Si–O absorption band was significant and became stronger in the presence of impurity (compare the values of *ν*_M_ ≈ 1076 cm^−1^ and *ν*_M_ ≈ 1086 cm^−1^ for annealed non-doped and doped oxides, respectively). These values of *ν*_M_ make it possible to conclude [16] that such treatment results in the average stoichiometry index value of “pure” oxide ~1.92, whereas in the presence of Er and F, this value was 2.0. At the same time on the spectra of both kinds of annealed films, the peak at ~810 cm^−1^ appeared. This band is usually connected with stretching symmetrical vibrations of oxygen atoms in silicon dioxide network [16, 19]. These facts enabled us to conclude that, due to annealing at 750 °C, a SiO_2_ phase is formed. In fact, the mathematical analysis of the main band shape showed that in the network of undoped oxide Si–O_3_–Si complexes are present along with SiO_4_ tetrahedra (Fig. 2). The network of the doped oxide consisted of only SiO_4_ tetrahedra. However, in both cases, SiO_4_ tetrahedra were interconnected in four- and sixfold rings (Fig. 2).

For the SiO_*x*_ < Er,F > film, both the IUAL–IUTL and the IUAL–ILNUAL five-parametric models provide almost the same and rather high simulation accuracy:

- MSE_min_ *=* 0.0905 deg^2^ at *n*_1_ = 1.876, *k*_1_ = 0.0052, *n*_2_ = 1.573, *h*_1_ = 858 nm, *h*_2_ = 44 nm.
- MSE_min_ *=* 0.1035 deg^2^ at *n*_1_ = *n*_2b_ = 1.881, *n*_2t_ = 1.439, *k*_1_ = *k*_2b_ = 0.0045, *k*_2t_ = 0, *h*_1_ = 836 nm, *h*_2_ = 64 nm.

This shows that the actual structure of the film corresponds to even more complicated model in which the refractive index value is ≈ 1.88 in the bulk of the film and decreases on the sigmoidal law from 1.88 at the ≈ 65-nm depths to ≈ 1.50 at the surface. Adequate description of the SiO_*x*_ film was obtained in the framework of the six-parametric ILNUAL–IUTL model at *n*_1b_ = 1.7355, *n*_1t_ = 1.687, *k*_1b_ = *k*_1t_ = 0.0070, *n*_2_ = 1.458, *h*_1_ = 896 nm, and *h*_2_ = 97 nm.

After heat treatment at *1000 °С*, the band shift was more significant than in the case of annealing at 750 °C (Fig. 1). This shift was more pronounced for the doped film so the value of *ν*_M_ became ~1094 and ~1098 сm^−1^ for the annealed undoped and doped films, respectively. Besides, predominant formation of sixfold SiO_4_ tetrahedra rings was observed (Fig. 2). If one takes into account that oxygen atoms oscillate in such rings with more frequencies than in fourfold ones, such a band shift may be explained only by structural changes in the oxide network. It should be also noted that the value of *ν*_M_ ≈ 1095 ± 5 сm^−1^ is typical for thick (~1 μm) films of the thermally grown silica in which the network namely sixfold rings of SiO_4_ tetrahedra dominate [12]. The 1000 °С annealed films are described in the framework of the two-layer models with the sharp boundary between the layers. For the SiO_*x*_ < Er,F > film, it is the UALNUTL–IUTL model at *n*_1bo_ = 1.855, *n*_1to_ = 1.787, *n*_1eb_/*n*_1ob_ = *n*_1et_/*n*_1ot_ = 1.002, *n*_2_ = 1.465, *h*_1_ = 813 nm, and *h*_2_ = 113 nm. For the SiO_*x*_ film, it is the UAUAL–IUTL model at *n*_1o_ = 1.684, *n*_1e_/*n*_1o_ = 1.002, *k*_1_ = 0.001, *n*_2_ = 1.472, *h*_1_ = 856 nm, and *h*_2_ = 157 nm. Small anisotropy can be due to the difference in thermal expansion coefficients of the substrate and the SiO_2_ matrix of the composite layer.

## Discussion

### As-Deposited Films

When comparing the properties of the as-deposited films, the attention is immediately drawn to the fact that, while the stoichiometry index value in the SiO_*x*_ and SiO_*x*_ < Er,F > films is almost the same, the latter film has lower refractive index value. It is clear that this is due to the peculiarities of its structure associated with the specifics of this film formation. The process of the SiO_*x*_ < Er,F > film formation has not been studied yet. Taking into account the similarity of the erbium and terbium fluorides’ thermodynamic properties [20], in order to understand the structural features of the as-deposited SiO_*x*_ < Er,F > film, it is appropriate to reference the mass spectroscopy data obtained during and after the deposition of SiO_*x*_ < Tb,F > films by co-evaporation of silicon monoxide and terbium fluoride [21, 22].

In the case of separate evaporation, the vapor over silicon monoxide consisted of the Si atoms and SiO molecules; terbium fluoride vapor had complex composition—Tb^+^, TbF^+^, TbF_2_^+^, TbF_3_^+^, F^+^, and HF^+^ ions have been found [21, 22]. In the case of co-evaporation of the monoxide and fluoride, the vapor-phase mass spectra also indicated the presence of SiF^+^, SiF_2_^+^, and SiF_3_^+^ ions. The exchange reactions took place in the solid-state phase during the film formation on the substrate as well. They provide the dissociation of TbF_3_ molecular complexes simultaneously with the Si–F and Tb–O chemical bond formation. The above described processes should decrease the stoichiometry index of the matrix in the doped film.

Apparently, all of the above is applicable to the SiO_*x*_ < Er,F > films. Accordingly, in our SiO_*x*_ < Er,F > films, Er and F can be incorporated into the as-deposited film not only in the form of ErF_3_ quasimolecular complexes but also in other configurations. Apparently, the decrease in the refractive index is caused by the influence of both fluorine and erbium atoms on the oxide. It is known that the introduction of fluorine leads to a less dense Si–O–Si network in fluorinated silicon oxide films than in SiO_2_ films [23]. This results in their lower refractive index than that of SiO_2_. The authors in [24] found the correlation between nanocrystal density, size, and the Er concentration in the 300–1300 °C annealed SiO_*x*_ < Er > films. This finding can be explained by considering Er as promoter for SiO_*x*_ disproportionation. Since oxygen shows the higher affinity for Er compared to Si, Er centers scavenge mobile oxygen atoms, thereby locally enhancing SiO_*x*_ disproportionation and consequently promoting Si nucleation near Er centers. In our case, such processes begin to appear during the formation of the SiO_*x*_ < Er,F > film on the substrate heated to 150 °C. The presence of the initial stage of phase separation in the SiO_*x*_ < Er,F > film is consistent with the red absorption edge shift (≈0.2 eV) in comparison with the undoped film. In this case, both the oxide matrix with higher stoichiometry index and the very small (≤1 nm) silicon clusters with *n* ~ 1.5 [25] formed in it have *n* values that are lower than the *n* value of the original homogeneous oxide. Thus, the concentration of silicon atoms that can form the silicon phase during the subsequent high-temperature treatment is higher in the as-deposited SiO_*x*_ < Er,F > film.

### Thermally Treated Films

To explain consistently the IR spectrometry and ellipsometry data for the heat-treated films, the following circumstances should be taken into account:

1. During thermal treatments on air (in the presence of oxygen and water vapor) of SiO_*x*_ films, two processes occur—thermostimulated phase separation, which leads to the formation of Si nanoinclusions embedded into SiO_*y*_ (*y > x*) matrix, and oxidation of silicon as well as silicon suboxide phases.
2. The process of phase separation is very rapid (seconds at 750 °C and less than 1 s at 1000 °C) [26] and proceeds much faster than the process of oxidation [27].
3. The process of oxidation should touch oxide matrix more efficiently than silicon inclusion, because in the latter case more Si–Si bonds (four per one silicon atom) are transforming into Si–O bonds.
4. In the case of silicon inclusions, oxidation rate decreases strongly when their size reduces [27, 28].
5. Annealing at 750 °C results in amorphous silicon nanoinclusion formation, whereas at 1000 °C, nanocrystalline phase dominates [29].
6. Er has direct beneficial impact on the conversion of SiO_*x*_ suboxide to elemental silicon and silica [24].
7. F enhances disorder-to-order transition in a-Si and its crystallization rate [30–32].
8. Oxidation of less ordered silicon and silicon suboxide is energetically more favorable because the strain energy generated by oxygen incorporation in the disordered material should be lower than the incorporation for the ordered one [33].

Hence, the following scenario of the processes taking part under high-temperature heat treatment in the air can be proposed.

*750 °C*. During the first few seconds of annealing, the phase separation of SiO_*x*_ takes place uniformly within the film volume. It results in the formation of amorphous nanoinclusions and the changes of oxide matrix stoichiometry—the stoichiometric index increases from ≈ 1.45 to ~1.7 (as data of heat treatments in pure argon had testified [29]). The oxide matrix, as in the case of annealing in argon, contains SiOSi_3_, SiO_2_Si_2_, SiO_3_Si, and SiO_4_ molecular complexes.

**Fig. 3:**
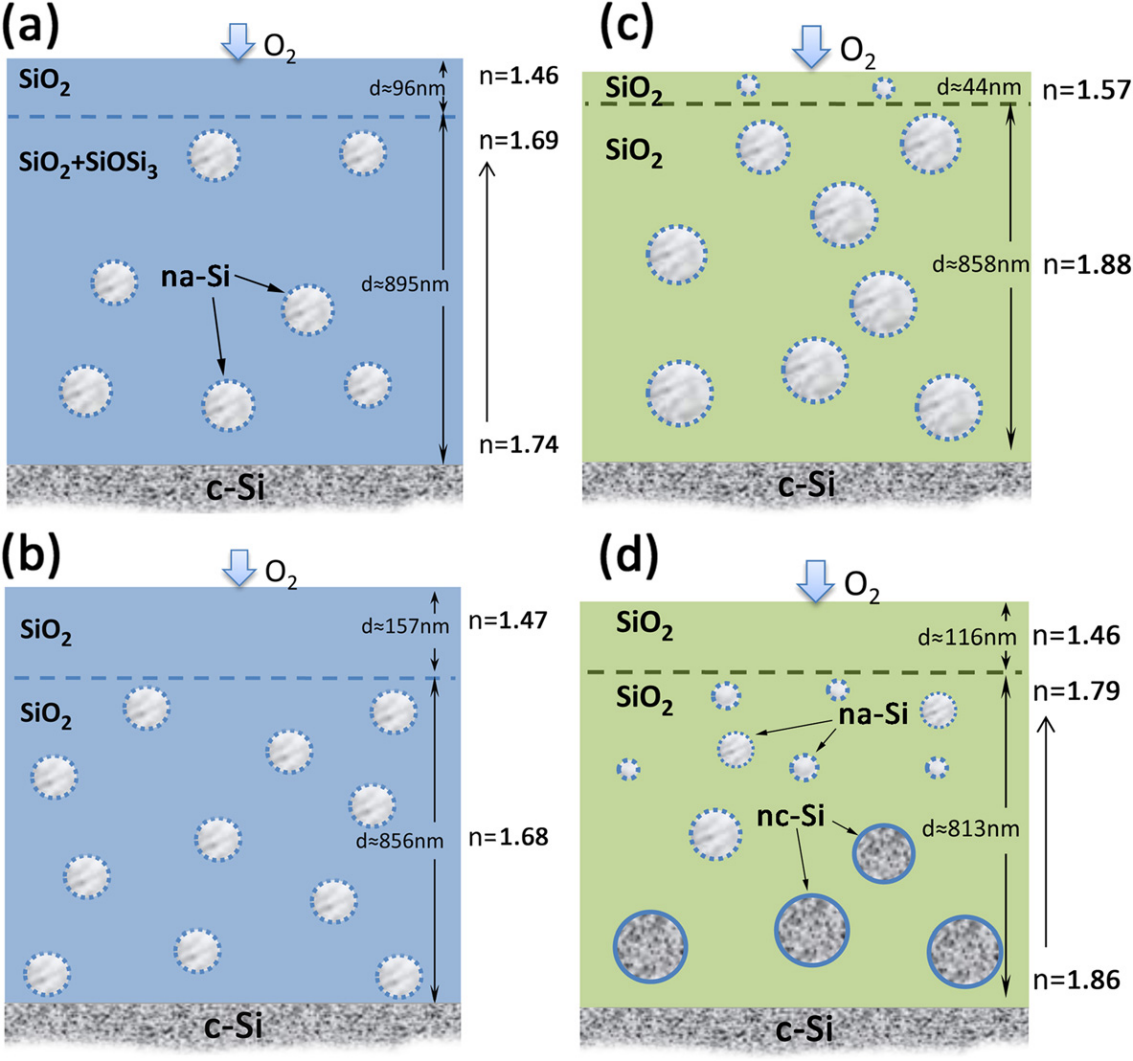
Schematic representation of nanocomposite films, obtained by annealing of the as-deposited films in air. **a** SiO_*x*_, *Т*_an_ = 750 °C; **b** SiO_*x*_, *Т*_an_ = 1000 °C; **c** SiO_*x*_ < Er,F>, *Т*_an_ = 750 °C; **d** SiO_*x*_ < Er,F>, *Т*_an_ = 1000 °C

Then, the oxidation of Si–Si bonds situated both on the silicon inclusion surface and in the oxide matrix occurs. The absence of the SiOSi_3_ and SiO_2_Si_2_ complexes shows that during 1-h annealing the effect of atmospheric oxygen is evident across the entire depth of the film. The intensity of oxidation depends on the distance from the film surface—it is much more pronounced within the outer layer of the film. As a result, in the case of the undoped oxide (Fig. 3a), the SiO_2_ layer free from silicon nanoinclusions is formed near the film surface. The deeper layer may be most likely represented as SiO_2_ matrix (SiO_4_ tetrahedra interconnecting in four- and sixfold rings) with some addition of SiO_3_Si complexes. In such a matrix, the nanoinclusions of amorphous silicon (na-Si) are embedded. Raman study confirmed the formation of the na-Si as a result of thermal treatment of SiO_*x*_ film at 750 °C in air [9, 10]. For the deeper layer, the *n* value slightly increases towards the substrate which can be related to the increase in the amount of the na-Si phase and SiO_3_Si complexes because of the weakening effect of oxygen when moving from the surface into the bulk of the film.

In the case of the SiO_*x*_ < Er,F > film, the processes occurring under annealing are generally the same, but the doping strongly influences on the outcome (Fig. 3b). It is known that the SiO_*x*_ films obtained by physical vapor deposition exhibit some porosity. This is due to the “soft” nature of the deposition process. Thermostimulated phase separation of the films is accompanied by a decrease in their porosity [11]. More pronounced densification of the annealed doped film is the first indication of the more significant rearrangements of its structure. According to the IR data, the matrix of the whole film is transformed from the suboxide into the dioxide. At the same time, the ellipsometry data testify that the dioxide matrix of the inner and outer parts of the film contains silicon nanoinclusions—in both parts, the value of *n* is significantly greater than that for SiO_2_. Moreover, the *n* value for the basic (inner) layer is also larger as compared to the undoped film. These facts may be explained if one assumes that the presence of Er and F impurities causes more complete decomposition of suboxide film and the formation of larger and more ordered silicon nanoparticles (the latter was confirmed by Raman data [10]). It seems quite natural that large enough na-Si inclusions are not fully oxidized during long-term heat treatment and some amount of size-reduced silicon particles remains even in the near-surface region of the film.

*1000 °C*. During the first seconds of annealing when the process of phase separation is prevailing, the structure of the SiO_*x*_ film is transformed similarly to the SiO_*x*_ films annealed at 1000 °C in inert atmosphere: the entire film represents amorphous and crystalline (up to 4 nm in size) silicon inclusions [34] in the inhomogeneous oxide matrix consisting of the mixture of the SiO_*y*_ (*y* < 2) and SiO_2_ (SiO_4_ tetrahedra bound into four-membered rings) phases [29]. More complete phase separation should be expected during this time in the SiO_*x*_ < Er,F > film.

Further heat treatment at such a high temperature should enhance the process of the Si–Si bond oxidation in both the matrix and on the surface of the Si particles. In fact, the structure of the matrix involves only SiO_4_ tetrahedra interconnecting in four- and sixfold rings even in the case of the undoped film (Fig. 2). The outer layer that is free from silicon inclusions is much thicker when compared with the film annealed at 750 °C (Fig. 3b, d). The inner layer may be represented as SiO_2_ matrix with both na-Si and nc-Si capsulated particles [10] in the case of the SiO_*x*_ < Er,F > film and na-Si particles only [10] in the case of the SiO_*x*_ film. The absence of nanocrystalline Si particles in the 1000 °C annealed SiO_*x*_ films may be explained if one assumes that oxidative action of air leads to structural transformations of silicon nanocrystals: the process of oxidation reduces the size of nc-Si inclusions and those of them with the size smaller than the critical value of ~2 nm [35] became amorphous. The concentration of the silicon crystalline phase formed in the first seconds of the heat treatment and the size of the silicon nanocrystals (≥7 nm [10]) are greater in the doped film; thus, the oxidation-stimulated amorphization of nc-Si proceeds more slowly (Fig. 3d).

## Conclusions

The air-annealed SiO_*x*_ and SiO_*x*_ < Er,F > films have a two-layer macrostructure similar to one of the Si/SiO_*x*_ nanocomposites formed in vacuum or inert atmosphere and subjected to additional annealing in an oxidizing atmosphere. The thermal treatment of non-stoichiometric SiO_*x*_ films produces the Si/SiO_*x*_ nanocomposites with more stoichiometric matrix at a lower temperature of the process as compared with annealing in a vacuum or inert atmosphere. Doping of SiO_*x*_ films with Er and F produces the nanocomposites with more perfect structure of both the Si nanoparticles and the oxide matrix. This explains the strong luminescence of erbium after heat treatment of SiO_*x*_ < Er,F > films at 750 °C [9].

## Abbreviations

FTIR: Fourier transform infrared spectroscopy
MSE: mean squared error
ITE: inverse task of ellipsometry
IUTL: isotropic uniform transparent layer
IUAL: isotropic uniform absorbing layer
UAUAL: uniaxially anisotropic uniform absorbing layer
ILNUTL: isotropic linearly non-uniform transparent layer
ILNUAL: isotropic linearly non-uniform absorbing layer
UALNUTL: uniaxially anisotropic linearly non-uniform transparent layer
IR: infrared
